# Nanoporous Gold and Other Related Materials

**DOI:** 10.3390/nano9081080

**Published:** 2019-07-27

**Authors:** Keith J. Stine

**Affiliations:** Department of Chemistry and Biochemistry, University of Missouri, Saint Louis, MO 63121, USA; kstine@umsl.edu; Tel.: +1-314-516-5346

The field of nanomaterials continues to expand with the discovery of new nanostructures opening up new possibilities for both the study of unique physical properties and new applications. Over the past 15 years, the study of nanoporous gold (np-Au) has accelerated rapidly, with the material initially referred to as porous gold. In 2004, there were 13 papers published in this area, while in 2018, the number was 119 with the total number of papers well over 1000. Some of the early work on porous gold has been reviewed [1,2]. It was quickly realized that np-Au posed high interest as a support material that could be modified with biological molecules and had the prospects for many applications in life science in biosensor development, drug release, and as a surface for controlled interactions with living cells. The material has many special properties that have spawned entire avenues of investigation, including novel plasmonic optical properties, catalytic properties, and mechanical properties. The study of nanoporous forms of other metals has also been stimulated by these discoveries, with gold having the special properties of its environmental and chemical stability. The entire field of nanostructured forms of gold and other metals or alloys continues to rapidly advance [3]. The modification of these nanomaterials has also stimulated a continuing development of many novel and more complex modified nanoparticles, especially for biological applications. This special issue covers a wide range of aspects of np-Au and closely related materials including biosensing, cellular interactions, and mechanical properties. Some aspects of novel developments of nanoparticles are also presented in this issue.

One of the most widely explored applications of np-Au has been to the field of biosensor development, as reviewed in this issue by Bhattarai et al. [4]. This nanoporous metal offers advantages of providing an inert gold surface that is readily surface modified using self-assembled monolayer technology or by the direct adsorption of biomolecules. The adjustable pore size can fall in a range that is especially suitable for the incorporation of proteins, oligonucleotides, antibodies, and enzymes within the structure. The applications of these np-Au based biosensors are very broad and cover the detection of DNA, cancer biomarker proteins, and many applications for sensing glucose. The development of biosensors based on np-Au continues to be an active and growing field [5,6,7]. In this issue, the exciting field of DNA detection based on np-Au is explored in the paper by Seker and coworkers [8]. The use of np-Au for electrochemical detection of DNA hybridization is shown to overcome some of the limitations in sensitivity, selectivity, and detection limits that are found when flat gold surfaces are used. Electrochemical biosensors built on np-Au for specific oligonucleotides that are biomarkers for breast cancer genes are demonstrated in a microfluidic format. The rising interest in biomarker detection based on DNA and for example on circulating DNA indicates that there is great potential for electrochemical biosensors based on np-Au in the rapidly growing field of precision medicine that is on the cusp of broad commercialization [9,10,11]. There is a need for nanostructured electrodes for use in electrophysiology studies and especially for the study of neural behavior and neurotransmitter levels in the brain [12]. The article in this issue by Seker and coworkers demonstrates that use of np-Au as an electrode material can reduce the problem of astrocyte adhesion to the gold surface through the change in morphology from that of a flat gold surface [13]. In this study, the new concept of a morphology library is introduced so that the variation of cellular adhesion with np-Au morphological features such as ligament dimensions and pore size can be studied. This paper also demonstrates the integration of np-Au, microfluidics, and cellular adhesion studies. Additional aspects of the applications of np-Au in life sciences, including promising applications such as controlled drug release [14,15], have recently been reviewed [16]. The basic mechanical properties of np-Au continue to be improved and the paper by Leitner and coworkers [17] presents a novel strategy to improve the yield strength of np-Au by plastic deformation of an AuFe nanocomposite followed by the etching of iron. There are many aspects of interest for np-Au, which is why it is a material of such high interest, and novel mechanical properties have been a major research direction [18,19]. The ability to form macroscopic size samples of np-Au enables their study by special nanoidentation methods, as are described in the paper. The interconnected morphology of ligaments and pores that is seen in np-Au is also found in other nanostructured metals, such as nanoporous copper (NPC) formed on the surface of the bulk metallic glasses (BMG) of the alloy Cu_50_Zr_45_A_l5_ by HF treatment, as studied in the paper by Wang and coworkers [20]. These rod like structures with a BMG interior and NPC shell are shown to exhibit superior energy absorption properties.

In addition to np-Au, there are other nanostructures of gold that can be made such as dendritic gold nano-forests, whose novel optical properties are used in the paper by Shiao and coworkers [21]. Gold nanostructures exhibit localized surface plasmon resonance behavior that can be tuned by varying the morphology [22]. Using the plasmon absorbance of these dendritic nano-forests, this study demonstrates their application as a photocatalyst for the degradation of pollutants. 

Two of the papers in this Special issue focus on nanoparticle structures targeted at biological applications. In the paper by Li and coworkers [23], a new approach to the preparation of polyion complex vesicles using gold nanoparticles as templates. Using block copolymers of opposite charge, vesicles are produced that could have applications in drug delivery and other areas and have tunable features that make them attractive alternatives to the traditional lipid based vesicles. The paper by Bigi and coworkers [24] describes the production of nanocrystals of hydroxyapatite that, when functionalized with hydroxystearate, show enhanced binding of silver nanoparticles that are widely under investigation for their antimicrobial properties [25].

I would like to thank all authors of this Special Issue of *Nanomaterials* for their contributions and the prompt and constructive comments from the referees, and also the consistently outstanding and efficient support from the editorial office.
